# Oral Health Status of Healthcare Workers in Ilembula/Tanzania during the COVID-19 Condition

**DOI:** 10.3390/healthcare12090920

**Published:** 2024-04-29

**Authors:** Tobias Bensel, Simon Megiroo, Werner Kronenberg, Wolfgang Bömicke, Timo Ulrichs, Sebastian Hinz

**Affiliations:** 1 Institute for Research in International Assistance, Akkon University for Human Sciences, Colditzstraße 34-36, 12099 Berlin, Germany; tobiasbensel@zahnarzt-am-rain.de (T.B.); timo.ulrichs@akkon-hochschule.de (T.U.); 2 Health Department, ELCT/North Central Diocese, Arusha P.O. Box 16173, Tanzania; s2003megiroo@yahoo.co.uk; 3 Ilembula Lutheran Hospital, Ilembula P.O. Box 14, Tanzania; werner-kronenberg@web.de; 4 Department of Prosthetic Dentistry, University of Heidelberg, Im Neuenheimer Feld 400, 69120 Heidelberg, Germany; wolfgang.boemicke@med.uni-heidelberg.de; 5 Department of Reconstructive Dentistry and Gerodontology, School of Dental Medicine, University of Bern, 3010 Bern, Switzerland

**Keywords:** caries incidence, Tanzania, general oral health, epidemiology, prosthetic treatment

## Abstract

The challenge of reduced dental treatment and education infrastructure in the Tanzanian highlands affects the oral health situation of both the general population and local healthcare workers. The aim of this study was to investigate the oral health status of healthcare workers at Ilembula Lutheran Hospital (ILH), Tanzania, during the COVID-19 pandemic. In total, 134 healthcare workers (62 women, 72 men; mean age 36.48 ± 9.56 years, range 19–59 years; median age 35.00 years) participated in this cross-sectional study, conducted from 12 February to 27 February. A dental examiner trained in oral health screening performed the oral health data collection. Data collection was performed by probability sampling using the Ilembula Data Collection Form—Oral Health (IDCF-Oral Health) questionnaire distributed in paper form. Ethical approval was obtained from the National Institute for Medical Research/Tanzania. The decayed, missing, and filled teeth (DMF/T) index proposed by the World Health Organization (WHO) was used with the associated caries measurement method and the simplified oral hygiene index (OHI-S). Details regarding edentulism, nutritional habits, and socio-economic factors were collected. Statistical analysis was performed using linear regression (α = 0.05). The average DMF-T index was 3.33 ± 0.82, with age, gender, meal frequency, and soft drink consumption significantly influencing the index. No evidence of dental plaque was detected in 43.3% of the participants. Of the participants, 32.8% required prosthetic treatment (Kennedy Class III), while 16.4% needed it for acute malocclusions. Oral hygiene products were used in 97% of cases. A total of 35.8% of the participants had an OHI-S score of up to 1.0, with (*p* < 0.001) age and (*p* < 0.001) sex having a significant influence on the index. The current oral health situation of healthcare workers at ILH shows a moderate need for restorative and prosthetic treatment in rural Tanzania. Despite the COVID-19 pandemic, there was no change in the need for dental treatment, which may be explained by the generally restricted access to dental healthcare in the investigated region. The development of an interdisciplinary oral health prophylaxis system could help to reduce the need for future treatments.

## 1. Introduction

Oral cavity diseases are the most common global diseases and are associated with social and behavioral factors. The most common oral diseases are caries and periodontitis [1]. The prevalence and severity of dental caries are still lower in the non-urban regions of East Africa than in industrialized Western countries [2]. The World Health Organization (WHO) recognizes that poor oral health, systemic diseases, inequality, and increased poverty are the major factors contributing to periodontal diseases [3]. Currently, the WHO estimates that approximately 50% of the global population (close to 3.5 billion people) suffer from a reduced oral health status [4].

Since the 1980s, there has been a rise in the consumption of refined sugar, which has increased the prevalence of dental caries in Tanzanian communities [5]. The number of oral health professionals per 10,000 people in Tanzania was 0.1 as of 2014–2019 [4].

An additional challenge in dental care is that the density of dental professionals differs greatly between urban and non-urban regions [2,6]. However, the limited number of dentists is not the only problem affecting dental care. In addition, the level of training of dental practitioners in non-urban areas is often lower than that in urbanized areas. This causes restricted access to high-quality healthcare services in rural parts of low-income nations, such as Tanzania [7]. Given this background, the observed continuous increase in the consumption of high-sugar foods must be viewed critically for the development of general oral health [8].

The implementation of community-based caries prevention programs in rural areas could sensitize the population to caries prophylaxis [9]. In principle, hospitals and hospital staff in non-urban areas are of particular importance as general points of contact for the population regarding health matters [10]. However, even in local health facilities, such as rural hospitals in Tanzania, there is a shortage of adequately qualified dental staff [5].

As the density of dental care is particularly low in non-urban regions of Tanzania, an increased incidence of caries with all its consequences, including tooth loss, is possible in the long term [1]. In Tanzania, dental treatment facilities, if they are available in non-urban regions, are often locally linked to existing hospital facilities [2]. This means that hospital facilities such as the Ilembula Lutheran Hospital (ILH) act as a point of contact for the population in the event of oral disease [10]. Given the lack of prophylaxis options available to the local population, it is important to keep an eye on the population’s oral health situation. As these hospitals are a point of contact for oral diseases, it is important to determine the level of individual oral health of healthcare workers, as they are seen as the logical point of contact by the population in the absence of specialist dental staff. Available data on the oral health status of general healthcare workers are very limited [2]. One recent study has been published on the oral health status of nursing staff in the non-urban regions of southwestern Tanzania [2].

At the time this study was conducted, the global COVID-19 pandemic was prevalent. Thus, this study was conducted under COVID-19 conditions [11]. The design of this study was such that this study could have been conducted at any time, and it was not directly related to the pandemic [12].

The current study aimed to assess the oral health status and the resulting conservative, surgical, and prosthetic treatment requirements of healthcare workers at a hospital in a non-urban area in southwest Tanzania. This study examined a closed cohort under COVID-19 conditions.

## 2. Materials and Methods

### 2.1. Participants

**Figure 1:**
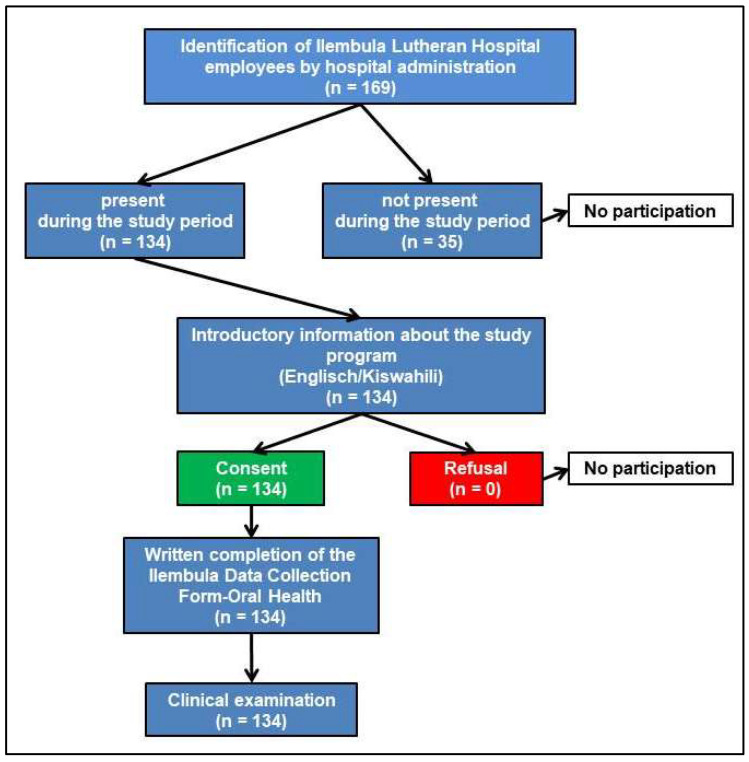
Schematic representation of the selection and participation process with the possibility of showing the voluntary nature of participation.

Of the 169 ILH healthcare workers (employees), 134 (62 women, 72 men; mean age 36.48 ± 9.56 years, range 19–59 years; median age 35.00 years) took part in this cross-sectional study during regular hospital operations and working hours. At the time this study was conducted, the hospital was understaffed, with 31 positions available. The participation rate of this study, in relation to the total possible participants, was 79%. There was a probability sample of ILH employees. Data were collected from 12 February to 27 February 2022, during the global COVID-19 pandemic. Data were collected using the probability sampling method. Written informed consent was obtained from all participants prior to participation. Approval for human research participants was obtained from the National Institute for Medical Research (NIMR), Tanzania (NIMR/HQ/R.8a/vol. IX/3192), and this study was conducted in accordance with the principles of the Declaration of Helsinki. The following inclusion criteria applied in order for participants to take part in this study: study participants had to be employees of the ILH and be on the hospital’s staffing list, which means that a maximum of 169 employees could have participated in this study; participation in this study took place during the employees’ regular working hours; and participants had to be of legal age. The subsequent exclusion criteria were defined: no persons under the age of 18 were allowed to participate in this study; no IIHAS employees or students were allowed to participate in this study; and no emeritus employees of the ILH were allowed to participate in this study. Pregnant employees were not excluded from participating in this study. Figure 1 illustrates the participation selection process which, if successful, was concluded with the clinical examination.

### 2.2. Study Site

The Evangelical Lutheran Church in Tanzania (ELCT), Southern Diocese is the owner of the ILH, a designated district council hospital that was first used as a health station in 1941 before being converted to a hospital in 1950. It is located in Tanzania’s southern Highlands in Ilembula village, Ilembula Ward, Wanging’ombe division of Wanging’ombe district, Njombe Region. The height of Ilembula is 1400 m above sea level. Up to 2.7 million people (the Morogoro–Mbyea region) commute from the Iringa region. With 317 beds, the ILH is one of the four hospitals in the area. Surgery, internal medicine, pediatrics, obstetrics, gynecology, radiography, laboratory, pharmacy, physiotherapy, laundry, dentistry, social welfare, nutrition and orphanage, rehabilitation units, tuberculosis/leprosy, clinical trial center, palliative care, reproductive and child health clinics, spiritual services, and funeral services are just a few of the healthcare services it provides. The ILH serves as a clinical medical, nursing, and midwifery teaching hospital for the Ilembula Institute of Health and Allied Sciences (IIHAS). In 2021, 28,701 patients were treated at ILH (Ilembula Lutheran Hospital Annual Report 2021).

### 2.3. Data Collection

The participants underwent an oral examination according to WHO guidelines and completed a questionnaire. The questionnaire was developed specifically for the Ilembula study area and was previously used in a study by Bensel et al. [2]. It was a hard copy questionnaire with the designation Ilembula Data Collection Form—Oral Health (IDCF-Oral Health), which was based on the guidelines for the design of case report forms [13]. The questionnaire had a closed structure. A bilingual (Kiswahili and English) study support administrator was available to answer any queries about the questionnaire or other aspects of this study in the participants’ native language. The data below were acquired via the questionnaire: (1) Personal data: age, sex, occupation; (2) medical and oral health status: general diseases, infectious diseases (e.g., HIV, tuberculosis, hepatitis), impaired general wound healing disorders, medication intake, and existing pregnancy (for women); (3) use of dental care products (e.g., use of a toothbrush, toothpaste and/or dental floss); and (4) social and diet behaviors (e.g., regularity and amount of consumption of coffee, tea, cigarettes, alcohol, and sugar-sweetened drinks).

### 2.4. Clinical Examination

This study involved a single examination of the oral cavity. Clinical examinations were performed by a dental examiner (TB) who was trained and experienced in oral health screenings [2]. The examination was carried out in the dental clinic of the ILH under the infrastructural conditions prevailing on site, using the lighting system of the existing dental unit. In accordance with the WHO recommendations for oral health screening, the instruments used included a dental mirror, dental probe, WHO probe, rubber gloves, and a face mask. All instruments that were used were disposable instruments, and after each person was examined, the disposable instruments were all disposed of. The decayed, missing, and filled teeth (DMF/T) indices were used to record the quantity of decayed, missing, and filled teeth. Caries was identified using the WHO criteria [14]. The simplified oral hygiene index (OHI-S) was used to evaluate oral hygiene and detect calculi and plaques. This was estimated for six tooth surfaces: the buccal surfaces were assessed in teeth 11, 16, 26, and 31, and the lingual surfaces were inspected in teeth 36 and 46 (according to the FDI World Dental Federation notation) [15]. The OHI-S categories and accompanying scores were as follows: good (0.0–1.2), moderate (1.3–3.0), and unfortunate (3.1–6.0). If present, the following intraoral attributes were described: medial diastema, gingival recession, and dental wear. The degree of wear was determined by the presence or absence of enamel surface features. Partial edentulism was characterized using the Eichner–Kennedy classification [16]. According to the Eichner classification, (partial) dentition is functionally divided into three groups according to the presence of support zones. The complete dentition has four support zones, and the anterior teeth are not considered. The support zone consists of two opposing pairs of teeth. The Kennedy classification is a topographic categorization that assesses each jaw individually. There are four main classes of dental arches: Class I represents bilaterally shorter rows of teeth; Class II represents unilaterally shortened rows of teeth; Class III represents an interrupted dental arch with one or more dental gaps; and Class IV represents an interdental gap in the anterior region that expands beyond the midline. The primary classes I–III are subdivided into three subgroups based on the number of gaps found: subgroup 1, with one extra interdental gap; subgroup 2, with numerous interdental gaps; and subgroup 3, with multiple interdental gaps and limited retained teeth. The effects of oral habits were recorded in the study participants, if they were present. Oral habits are behavioral habits relating to the mouth that can lead to damage to the teeth and jaws (such as thumb or finger sucking, nail-biting, and bruxism). During the clinical examination, the teeth, oral cavity, and both jaws were examined. Dental dam presence, and the presence of intraoral abnormalities such as crossbite, tooth position anomalies, dental trauma, recession, gingival hyperplasia, candidiasis and mucosal diseases, was recorded.

### 2.5. Data Analysis

All statistical analyses were performed using IBM SPSS Statistics for Windows, Version 29.0.2.0 Armonk, NY, USA: IBM Corp. Released 2023. Linear regression was used to analyze the factors associated with the DMF/T and OHI-S scores. Differences were considered statistically significant at a *p*-value < 0.05.

## 3. Results

### 3.1. General Medical History and Dental Medical History

General medical history was unremarkable in the vast majority of study participants (*n* = 92, 68.7%). Almost all of the participants used their own oral hygiene products (*n* = 130, 97.0%) (Table 1).

A minority of the healthcare workers reported intraoral symptoms during the use of dental care products (*n* = 52, 38.8%) (Table 1). Previous dental treatments received in the past included direct fillings, tooth extractions, and dental checkups. Fifty-eight (44.3%) participants reported receiving dental treatment within the past 2 years (Table 1).

### 3.2. Social and Diet Behaviours

Most participants (*n* = 88, 65.7%) reported eating three meals per day. Forty-four (32.8%) participants did not eat sweet foods (Table 2). However, 128 (95.5%) participants reported a daily consumption of sugar-sweetened tea. Ninety-two participants (68.7%) reported a daily consumption of soft drinks (Table 2). Only 16 participants (11.9%) reported alcohol consumption. The consumption of tobacco products was even lower (4.5%, *n* = 6) (Table 2). Taken together, tobacco and alcohol consumption were very low according to the participants, as presented in Table 2.

### 3.3. Oral Health Data

**Figure 2:**
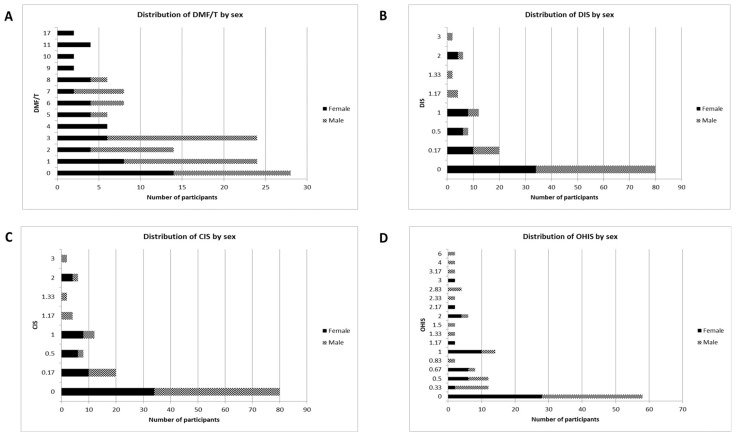
Value of (**A**) DMF/T (decayed, missing, and filled teeth), (**B**) DIS (debris index score), (**C**) CIS (calculus index score), and (**D**) OHIS (oral hygiene index score).

**Figure 3:**
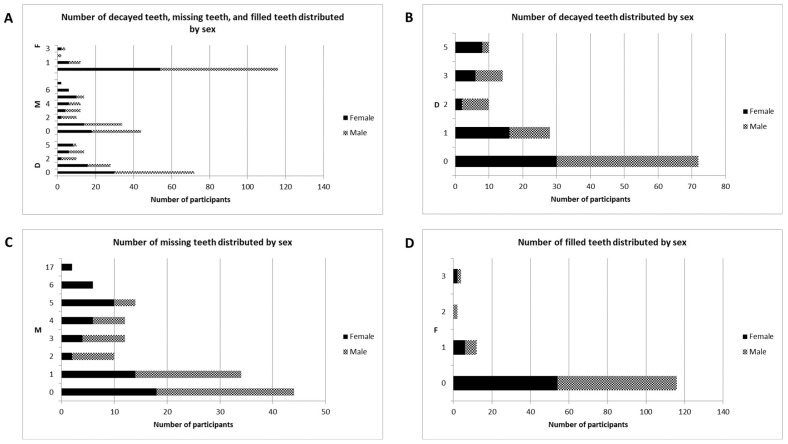
Distribution of the number of filled, missing, and decayed teeth by sex (**A**): decayed teeth (**B**), missing teeth (**C**), and filled teeth (**D**).

The mean DMF/T index of the participants was 3.33 ± 0.29 (Table 3). The incidence of caries in almost half of the participants (*n* = 52, 38.8%) was very low (DMF/T index, 0–1) (Figure 2A and Figure 3A). The majority of the participants showed a very low debris index score of 0.00 (53.7%) (Figure 2B). This was also true for the calculus index score of 0.00 (59.7%) (Figure 2C), and the oral hygiene index score of 0.00 (43.3%) (Figure 2D). No carious lesions were observed in 53.7% of the study participants (Figure 3B). The majority of the participants (*n* = 90, 67.2%) had missing teeth (M) (Figure 3C). Only 13.4% of the participants had at least one filled tooth (Figure 3D). Age (*p* < 0.001), sex (*p* < 0.001), number of meals per day (*p* < 0.001), and soft drink consumption per day (*p* = 0.045) significantly influenced the DMF/T index (Table 4). Around 48 participants (35.8%) showed an OHI-S score up to 1.0 (Table 5). The age (*p* = 0.043) and sex (*p* = 0.003) of the study participants had a significant influence on OHI-S scores (Table 5). Regarding dental status, 106 of the study participants (79.1%) had at least Eichner class A2 (Table 6). The need for prosthetic treatment in both the maxilla (*n* = 22, 16.4%) and mandible (*n* = 24, 17.9%) was most frequently due to the presence of Kennedy class III (Table 6). The most common carious and missing teeth were the second molars of the maxilla (Table 7).

### 3.4. Tooth Position Anomalies

Most participants (*n* = 86, 64.2%) exhibited regular tooth positions. Midline diastema (*n* = 28; 20.9%) was the most common deviation from the norm. The other tooth position anomalies identified in the participants are listed in Table 8.

## 4. Discussion

This research included 134 healthcare workers from ILH. The sexes were evenly represented. This was unexpected, given that the majority of practicing healthcare workers in Tanzanian villages are women [6]; however, it bolstered the investigation into sex-specific dependence.

None of the participants had suffered from a SARS-CoV-2 infection or COVID-19 disease in the past 12 months [17]. It should be noted that recent SARS-CoV-2 infection or COVID-19 disease was only queried anamnestically. No tests were carried out regarding the pathogen in relation to this study. The interpretation of this result means that a connection to the global pandemic cannot be made, as this study was only conducted under COVID-19 conditions. Thus, this study does not provide any objective evidence of an effect of a SARS-CoV-2 infection or COVID-19 disease on oral health. The majority of participants (*n* = 92, 68.7%) stated they were free of general diseases. This may be related to the general medical background of the research participants, who may have been focused on maintaining improved general personal health. However, the high general health ratings could also be explained by the anamnestic data collection technique. This may be especially true for the spread of infectious diseases. The proportion of HIV-positive participants was much lower than Tanzania’s average infection rate (0% vs. 4.8%) [18]. A total of 4.5% of the surveyed participants took medications. Questions about the usage of specific drugs were not asked.

The majority of the healthcare workers who took part in this study (*n* = 94; 70.1%) used toothpaste and owned their own toothbrushes (*n* = 76; 56.1%) [19,20]. The research did not inquire as to whether the toothpaste contained fluoride as an active component. The majority of healthcare workers cleaned their teeth twice a day (*n* = 76; 56.7%). Male healthcare workers were more likely than female healthcare workers to clean their teeth more than twice per day (0.75% vs. 0.70%) [21,22]. The differences between the sexes were minimal and insignificant. In general, women demonstrated higher health awareness than men, which might be regarded as reflective of the individual use of dental care products [23]. Interdental cleaning was performed by only a few participants [2,6]. This would require increasing the density of dentists, not only throughout Tanzania but especially in non-urban areas, such as Ilembula, to counsel adults with individualized oral health instructions. Almost two-thirds of the participating healthcare personnel stated they had not had an acute toothache at the time of the inquiry. These results imply that toothache was an irrational complaint. Additionally, societal and cultural factors influence how people perceive pain [24]. Gum bleeding was reported in a small number of participants (*n* = 8, 6.0%) [25]. More male healthcare workers suffered from gum bleeding than their female counterparts. This may be because men are less effective than women at providing home-based oral care [23]. Misuse of the toothbrush is a typical cause of increased gingival bleeding during home dental care. The use of broken or outdated toothbrushes can exacerbate gum disease, even if the DMF/T index is low [25]. Older healthcare workers showed increased susceptibility to periodontal disease. This finding is consistent with observations in Western industrialized countries, where the prevalence of periodontal disease is correlated with age [26,27].

More than half of the participants stated that they had not undergone a dental checkup in the last 2 years [28]. Tooth extraction was the most commonly performed treatment [29]. However, this frequent form of treatment had been administered to only 7.4% of the participants in the last 2 years. Nearly one in five participants had undergone a dental examination two years prior to the study visit. This is half the average for other parts of Tanzania, where approximately 40% of the respondents had annual dental checkups [19]. The main reason for the low frequency of dental consultations was the very low density of fully trained dentists in Tanzania, with a ratio of 0.1 dentists per 10,000 people in the current WHO Oral Health Country Profile [30]. This figure is even lower in non-urban regions, such as Ilembula [7]. Therefore, the majority of the Tanzanian population primarily seeks dental care for pain management [28,31,32].

Most of the study participants (*n* = 88, 65.7%) ate three meals per day [25]. The consumption rate of sweet beverages, sugar-sweetened tea, and soft drinks was similar to that of the average adult population in rural areas [25]. In East Africa, sugar-sweetened food consumption was greater in cities than in rural regions [5,33]. Increased daily intake of soft drinks, sugar-sweetened tea, and sweet beverages has been associated with poor oral health [5,34]. In this study group, alcohol and tobacco use was much lower than the Tanzanian average [20]. This may be a result of Tanzania’s ban on smoking in public places and the rigorous prohibition of drinking alcohol on ILH property. Another possible explanation for this finding is that the research participants were healthcare workers, making them more aware of the health risks associated with alcohol and tobacco use [35,36].

The overall prevalence of caries in the study population was *n* = 62 (46.3%). Compared to previous studies of dental caries in adult Tanzanians, this was significantly higher [30,37] Nonetheless, compared to nations with greater incomes, the frequency of dental caries was lower, as indicated by the DMF/T index [34]. The research participants’ overall mean DMF/T index agreed with the results of other adult Tanzanian populations that have been published [28]. Therefore, the DMF/T index scores in this study were based on the increased number of missing teeth.

It is not uncommon for non-urban regions in countries with a low gross domestic product (GDP) to have a higher proportion of adults with missing teeth that have not been prosthetically restored. A low dental density results in inadequate conservative care, which usually leads to tooth loss [30,38]. Furthermore, caries was discovered visually in this investigation. Further dental testing or radiography might provide a more complete description of the degrees of individual caries cases and individual treatment needs. Caries risk is often correlated with the kind and frequency of sugary food consumption, with increased frequency being associated with an increased risk of caries [39]. The prevalence rate of caries has been linked to rising sugar consumption in low-income nations, and sugar consumption is rising in Africa [34,38]. Consequently, it is not only the younger generation that consumes sugary foods. The industry’s general advertising campaigns portray the Western lifestyle imitated by many East African population groups [34,40].

Although not as excellent as that reported in other rural locations, the current study’s observations of personal oral hygiene were nonetheless good [6]. This result could, however, be explained by the research participants’ greater mean age. In the current investigation, soft plaques were observed in both men and women. This resulted in high simplified debris index (DI/S) ratings, which contributed to the overall OHI/S scores. Knowledge of personal oral hygiene is reduced in the wider population of Tanzania [6]. Nonetheless, it may be presumed that the research participants participated in Tanzania’s primary school oral health program, which was adopted by the government in 1982 [19]. It was thus anticipated that research participants would be familiar with the fundamentals of oral health. Low DMF/T index values in the worldwide comparison indicated good oral hygiene among the research participants [5]. The effects of personal oral hygiene should be documented in future research, using a coloring solution to depict the participants’ soft plaque regions.

The incidence of midline diastema in a population varies depending on the study region [41]. In the present study, midline diastema of the upper jaw was recorded in one-fifth of the participants (*n* = 28; 20.9%). Midline diastema with a hereditary component, in the absence of other oral malformations and oral health limitations, does not require dental or surgical treatment [41]. Nearly one in five subjects (*n* = 30; 22.4%) showed gingival recession, with a higher percentage in men [42]. Gingival recession may be related with dental calculus and plaque, increasing the risk of periodontal disease [42]. This study’s subjects maintained acceptable personal oral hygiene. Due to their poorer overall personal oral hygiene, this may have contributed to the typically low incidence of gingival recession in this research, and the higher prevalence of gingival recession in males.

Average tooth loss was high among research participants. At least one tooth—including the wisdom teeth—was missing in almost two-thirds (*n* = 90; 67.2%) of the participants. The similarity of these outcomes is contingent upon the research participants’ median age [32]. Consequently, it is anticipated that the number of missing teeth will rise as the population’s median age rises, and as eating patterns potentially change in the future [1,6,20]. Single-tooth gaps (Kennedy class III) were detected in most of the research participants [1,22,43]. The mandible was more commonly identified as the primary partly edentulous arch. Kennedy class IV was not found in this study, which might be attributable to the participants’ relatively youthful median age compared to Western developed countries [43]. Tooth loss occurred roughly similarly in both sexes [20]. This also applied to women’s DMF/T index scores when compared to men’s. Untreated carious lesions are the basis for an elevated DMF/T index score, which might lead to early personal tooth loss. In Tanzania’s rural communities, tooth extractions are the most prevalent dental treatment [1,2]. Generally, healthcare workers face greater and more stressful workloads during a pandemic [44]. Therefore, it could be assumed that the oral health of health workers was neglected during the pandemic, and that a higher incidence of oral health-related diseases could be observed in this cohort. However, this statement cannot be made in this descriptive study. The data did not differ noticeably from the general oral health data of adults [45], and there are currently no comparative data available on the results of this descriptive study.

This study focused on healthcare personnel at the ILH in Ilembula, Tanzania, which might be the main limitation of this study. This study was performed in a hospital in a rural region without any randomization. Consequently, it should be taken into account that there is no discernible difference between the oral health status of the general population and that of rural or urban areas. Furthermore, because healthcare professionals may have different socioeconomic origins, educational backgrounds, and access to medical and dental treatments than the general public, it might be difficult to extrapolate this study’s findings to the oral health conditions of the surrounding community [2]. Therefore, the promotion of oral hygiene from an early age, both by school systems and within the family, may be the greatest and least expensive method for lowering the worldwide burden of dental illnesses, as self-care and regular dental hygiene are the most effective variables in avoiding dental diseases. This statement also applies to healthcare workers at the ILH, as most of them come from non-urban regions and were not able to participate in a structured oral health prophylaxis program during their youth. A further limitation of this study is that no testing for the SARS-CoV-2 virus was carried out on the study participants, and a connection to the pandemic could only be established anamnestically. During the global COVID-19 pandemic, there have been massive restrictions on regular health care [12]. Nevertheless, this study was performed in February 2022, during the worldwide COVID-19 pandemic situation.

## 5. Conclusions

It is evident that the majority of the ILH healthcare staff members had low DMF/T index scores and practiced appropriate oral hygiene. Recall trips to dental care facilities were not recorded with much frequency. However, the usage of personal oral health products and the practice of regular oral hygiene were higher than predicted. Because of this study’s methodology, no conclusion can be drawn as to whether the COVID-19 pandemic had an influence on participants’ oral health. Given that the majority of Tanzanian people seek dental treatment for pain relief, it is essential to raise awareness among professional healthcare providers in rural regions about the prevention of oral diseases. This study could be useful in establishing baseline data on global oral health. This is consistent with WHO oral health objectives. Overall, worldwide plans to enhance oral health, while adequate for most industrialized nations, may be less realistic for countries with smaller GDPs, particularly from a financial and logistical standpoint.

This study should be territorially extended to other rural areas in Tanzania or East Africa because of the lack of similar studies.

## Figures and Tables

**Table 1:** Distribution of the general medical and oral health status of healthcare workers.

Parameters	*n*		%
Sex			
Female	62		46.3
Male	72		53.7
Total	134		100.0
Medical History	Female	Male	
None	50	42	68.7
Known diseases	0	6	4.5
General wound healing disorders	2	0	1.5
Medication	4	2	4.5
Smoking in the last week	0	2	1.5
Alcohol	2	12	10.4
Oral habits	0	2	1.5
Smoking and alcohol	0	2	1.5
Known diseases and medication	4	4	6.0
COVID-19 reported in the last 12 months	0	0	0
Total	62	72	100.0
Dental care products			
None	2	2	3.0
Toothbrush	8	10	13.4
Toothbrush and toothpaste	34	42	56.7
Toothbrush, toothpaste, and toothpick	12	14	19.4
Toothbrush and toothpick	6	4	7.5
Total	62	72	100.0
Brushing teeth per day			
0	4	4	6.0
1	14	14	20.9
2	34	42	56.7
3	10	12	16.4
Total	62	72	100.0
While brushing			
None	34	48	61.2
Toothache	16	6	16.4
Gum hurt	2	6	6.0
Gum bleed	2	6	6.0
Toothache and gum hurt	4	0	3.0
Toothache and gum bleed	2	0	1.5
Gum hurt and gum bleed	0	2	1.5
Toothache, gum hurt, and gum bleed	2	4	4.5
Total	62	72	100.0
Dental treatment			
None	32	44	56.7
Check-up (2 year)	12	16	20.9
Extraction of a tooth (2 year)	10	0	7.5
Filling (2 year)	0	2	1.5
Other reason (2 year)	0	2	1.5
Check-up and tooth extraction	4	6	7.5
Check-up, tooth extraction, and filling	2	0	1.5
Check-up, tooth extraction, and other	2	0	1.5
Check-up and filling	0	2	1.5
Total	62	72	100.0
Crossbite			
No	54	62	86.6
Yes	8	10	13.4
Total	62	72	100.0
Dental trauma			
No	62	70	98.5
Yes	0	2	1.5
Total	62	72	100.0
Recession			
No	50	54	77.6
Yes	12	18	22.4
Total	62	72	100.0
Gingival hyperplasia			
No	62	68	97.0
Yes	0	4	3.0
Total	62	72	100.0
Candidiasis			
No	62	72	100.0
Yes	0	0	0.0
Total	62	72	100.0
Mucosal diseases			
No	62	68	97.0
Yes	0	4	3.0
Total	62	72	100.0

**Table 2:** Distribution of healthcare workers’ comments about their consumption of food and beverages.

Parameters	*n*		%
Smoking last week	Female	Male	
No	36	34	52.2
Never	26	32	43.3
Occasionally	0	4	3.0
Every day	0	2	1.5
Total	62	72	100.0
Alcohol			
No	60	58	88.1
Yes	2	14	11.9
Total	62	72	100.0
Meals per day			
1	2	2	3.0
2	14	28	31.3
3	46	42	65.7
Total	62	72	100.0
Sweets per day			
0	14	30	32.8
1	40	24	47.8
2	4	12	11.9
3	4	4	6.0
5	0	2	1.5
Total	62	72	100.0
Sugar-sweetened tea per day			
0	4	2	4.5
1	48	62	82.1
2	6	6	9.0
3	2	2	3.0
4	2	0	1.5
Total	62	72	100.0
Soft drinks per day			
0	20	22	31.3
1	36	34	52.2
2	6	16	16.4
Total	62	72	100.0

**Table 3:** Participants’ caries experience: decayed, missing, and filled teeth Index.

Parameters		DMF/T	DIS	CIS	OHIS
Mean		3.33	0.4403	0.3333	0.7736
Standard error of the mean		0.294	0.06281	0.05332	0.09833
Median		3.00	0.0000	0.0000	0.3333
Standard deviation		3.409	0.72703	0.61721	1.13820
Minimum		0	0.00	0.00	0.00
Maximum		17	3.00	3.00	6.00
Percentile	25	1.00	0.0000	0.0000	0.0000
	50	3.00	0.0000	0.0000	0.3333
	75	5.00	0.5000	0.5000	1.0000

DMF/T = decayed, missing, and filled teeth; OHIS = oral hygiene index score; DIS = debris index score; CIS = calculus index score.

**Table 4:** Factors related to the participants’ DMF/T based on a linear regression analysis.

DMF/T						
R^2^ = 0.382	Regression Coefficient B	SD	Beta	T	Sig.	95% Confidence Interval (CI) for B (Min–Max)
Constant	8.829	1.966		4.491	<0.001	4.939–12.720
Sex	−1.747	0.494	−0.256	−3.533	<0.001	−2.725–−0.768
Age	0.127	0.025	0.356	4.979	<0.001	0.076–0.177
Brushing teeth per day	−0.612	0.344	−0.138	−1.780	0.077	−1.292–0.068
Meals per day	−1.814	0.476	−0.289	−3.814	<0.001	−2.756–−0.873
Sweets per day	0.263	0.268	0.074	0.983	0.328	−0.267–0.792
Sugar–sweetened tea per day	−0.348	0.439	−0.062	−0.792	0.430	−1.218–0.522
Soft drinks per day	−0.794	0.393	−0.158	−2.020	0.045	−1.571–0.016

DMF/T = decayed, missing, and filled Teeth.

**Table 5:** Factors related with participants’ OHI-S based on a linear regression analysis.

OHIS						
R^2^ = 0.171	Regression Coefficient B	SD	Beta	T	Sig.	95% Confidence Interval (CI) for B (Min–Max)
Constant	−0.140	0.760		−0.184	0.854	−1.644–1.364
Sex	0.390	0.191	0.171	2.040	0.043	0.012–0.768
Age	0.030	0.010	0.248	3.003	0.003	0.010–0.049
Brushing teeth per day	−0.148	0.133	−0.100	−1.112	0.268	−0.411–0.115
Meals per day	−0.085	0.184	−0.040	−0.460	0.646	−0.448–0.279
Sweets per day	−0.091	0.103	−0.077	−0.879	0.381	−0.296–0.114
Sugar–sweetened tea per day	0.286	0.170	0.153	1.686	0.094	−0.050–0.623
Soft drinks per day	−0.137	0.152	−0.081	−0.901	0.369	−0.437–0.164

OHIS = oral hygiene index score.

**Table 6:** Distribution of dental status.

Parameters	*n*		%
Dental Status without Wisdom Teeth	Female	Male	
Full dentition	18	26	32.8
Partially edentulous	44	46	67.2
Total	62	72	100.0
Eichner classification			
None	14	14	20.9
A1	4	14	13.4
A2	14	20	25.4
A3	10	12	16.4
B1	14	12	19.4
B2	4	0	3.0
B4	2	0	1.5
Total	62	72	100.0
Maxilla—Kennedy classification (wisdom teeth included)			
None	28	44	53.7
Class I/1	2	0	1.5
Class II	10	4	10.4
Class II/1	2	0	1.5
Class III	4	18	16.4
Class III/1	10	6	11.9
Class III/2	6	0	4.5
Total	62	72	100.0
Mandible—Kennedy classification (wisdom teeth included)			
None	24	36	44.8
Class I	6	4	7.5
Class I/2	2	0	1.5
Class II	8	2	7.5
Class II/1	4	4	6.0
Class II/2	0	2	1.5
Class III	6	18	17.9
Class III/1	12	6	13.4
Total	62	72	100.0

**Table 7:** Number of decayed teeth, missing teeth, and filled teeth distributed by teeth.

Teeth (FDI)	Existing		D		M		F	
	*n*	%	*n*	%	*n*	%	*n*	%
18	88	65.7	8	6.0	38	28.4	0	0.0
17	108	80.6	12	9.0	12	9.0	2	1.5
16	108	80.6	10	7.5	12	9.0	4	3.0
15	114	85.1	4	3.0	16	11.9	0	0.0
14	116	86.6	4	3.0	14	10.4	0	0.0
13	130	97.0	0	0.0	4	3.0	0	0.0
12	132	98.5	2	1.5	0	0.0	0	0.0
11	126	94.0	6	4.5	2	1.5	0	0.0
21	124	92.5	6	4.5	4	3.0	0	0.0
22	128	95.5	4	3.0	2	1.5	0	0.0
23	128	95.5	2	1.5	4	3.0	0	0.0
24	122	91.0	4	3.0	8	6.0	0	0.0
25	116	86.6	8	6.0	10	7.5	0	0.0
26	104	77.6	8	6.0	18	13.4	4	3.0
27	96	71.6	20	14.9	18	13.4	0	0.0
28	84	62.7	12	9.0	38	28.4	0	0.0
38	82	61.2	14	10.4	36	26.9	2	1.5
37	78	58.2	16	11.9	30	22.4	10	7.5
36	92	68.7	10	7.5	32	23.9	0	0.0
35	122	91.0	2	1.5	6	4.5	4	3.0
34	134	100.0	0	0.0	0	0.0	0	0.0
33	132	98.5	2	1.5	0	0.0	0	0.0
32	134	100.0	0	0.0	0	0.0	0	0.0
31	130	97.0	2	1.5	2	1.5	0	0.0
41	130	97.0	2	1.5	2	1.5	0	0.0
42	132	98.5	0	0.0	2	1.5	0	0.0
43	134	100.0	0	0.0	0	0.0	0	0.0
44	130	97.0	0	0.0	4	3.0	0	0.0
45	124	92.5	2	1.5	8	6.0	0	0.0
46	90	67.2	6	4.5	36	26.9	2	1.5
47	86	64.2	14	10.4	32	23.9	2	1.5
48	92	68.7	4	3.0	38	28.4	0	0.0

D = decayed; M = missing; F = filled teeth.

**Table 8:** Tooth position anomalies.

Tooth Position	*n*		%
	Female	Male	
Normal	42	44	64.2
Midline diastema	12	16	20.9
Edge-to-edge bite	0	4	3.0
Anterior open bite	4	0	3.0
Anterior crowding	0	2	1.5
Nonocclusion	2	2	3.0
Anterior tooth trauma (maxilla)	0	2	1.5
Midline diastema and anterior open bite	2	0	1.5
Edge-to-edge bite and anterior crowding	0	2	1.5
Total	62	72	100.0

## Data Availability

The data used to support the findings of this study may be released upon an application to the Department of Reconstructive Dentistry and Gerodontology, School of Dental Medicine, University of Bern, which can be contacted through Sebastian Hinz, Department of Reconstructive Dentistry and Gerodontology, School of Dental Medicine, University of Bern, 3010 Bern, Switzerland.
